# “A place at the table:” a qualitative analysis of community board members’ experiences with academic HIV/AIDS research

**DOI:** 10.1186/s12874-016-0181-8

**Published:** 2016-07-11

**Authors:** Stella Safo, Chinazo Cunningham, Alice Beckman, Lorlette Haughton, Joanna L. Starrels

**Affiliations:** Montefiore Medical Center, 111 E 210 St, Bronx, NY 10467 United States; Department of General Internal Medicine, New York, USA; Albert Einstein School of Medicine, New York, USA

**Keywords:** Community based participatory research, Community advisory boards, HIV/AIDS, Academic-community partnerships

## Abstract

**Background:**

Community advisory boards (CAB) are proposed as one mechanism to carry out successful community based participatory research (CBPR), but the presence of CABs may be insufficient to optimize academic-community partnerships.

**Methods:**

We conducted semi-structured interviews with minority members of a CAB partnered with a HIV/AIDS research center and identified three themes.

**Results:**

First, lack of trust in researchers included two subthemes: researchers’ lacked respect for community-based organizations’ (CBO’s) interests and paid inadequate attention to building trust. Second, power imbalance included three subthemes: CAB members felt like inferior “token” members, felt that a lack of communication led to disempowerment, and held preconceived beliefs of researchers that led to perceived power imbalance. Third, CAB members suggested best practices, including using collaborations to build trust, actively allocating power, and sharing tangible research benefits with CBOs.

**Conclusions:**

Our findings indicate that CABs must be founded on trust and instilled with power to meet the tenets of CBPR.

## Background

Ideal community based participatory research (CBPR) involves community members at every step, from the inception of the study question to the dissemination of findings [1–4]. However, there remains a gap between ideal CBPR and its real-world application [1, 2, 5]. In actuality, the extent of community members’ involvement in CBPR is often limited; for example, involving community members only to help recruit study participants [5, 6]. Community advisory boards (CABs) — boards comprised of members of a community who collaborate with academic researchers on research endeavors — have been proposed as a solution to achieve a more equitable, effective, and engaged academic-community partnership [7, 8]. The field of HIV/AIDS research has long involved CABs, in part because community activists worked with the National Institute of Allergy and Infectious Disease (NIAID) to establish a 1990 mandate that all NIAID-funded research establish CABs [7, 9, 10]. Since the NIAID mandate, several studies have examined how CABs can be used to support ideal CBPR in HIV/AIDS research [5, 7, 11].

The limited research on HIV/AIDS CABs in the United States has focused mainly on the procedural function of these groups, examining which elements have helped or hindered the establishment of a successful CAB. Previous studies include explorations of CAB structures that have led to successful community partnerships [1, 11], case studies of exemplary CABs in different environments including those paired with academic centers [7, 12, 13], and surveys of CAB members and/or researchers to understand their attitudes and beliefs towards participating in a CAB [9, 10]. Findings from these studies indicate that successful CABs operate on transparency and equal participation from researchers and community members [12, 14] and set terms of engagement prior to the initiation of the partnership [1, 15]. Studies have also described numerous challenges that CABs face, including tensions that arise between researchers and community members due to disparities in training and access to resources [11, 16], unmatched priorities [17–19], lack of trust or respect [14, 20], and historical or structural racism [16, 21–23].

Apart from this focus on the functioning of HIV/AIDS CABs, there remains more to be learned about the nature of CABs as a tool to further participatory research in the field of HIV, particularly among minority communities [7, 24]. To our knowledge, previous studies have not conducted in-depth qualitative analyses of an urban HIV-focused CAB comprised of community members who work with an underserved population of racial and ethnic minorities. This focus is particularly important because the burden of HIV infection disproportionately affects racial and ethnic minorities [25–27]. In addition, partnerships between communities of color and academic centers have been mired by a complex history that has included periods of mistrust [21, 24, 28], making a focus on these CABs important. To help address this gap, we sought to examine the challenges to academic-community partnerships in an urban and impoverished setting by exploring CAB members’ experiences in interacting with researchers and by identifying opportunities, or best practices, to improve these relationships.

## Methods

### Setting and participants

The Center for AIDS Research (CFAR) was created in 2003 within a major academic medical center in the Bronx, New York, to support and coordinate research efforts of NIH-funded HIV/AIDS research in the Bronx. The Bronx contains the poorest congressional district in the United States. It is comprised primarily of black and Latino residents and has prevalence rates of HIV/AIDS that are among the highest in New York City [29, 30]. In 2012, the CFAR community advisory board (CFAR CAB) was transformed from one focused on HIV care to one focused on HIV research [31]. Researchers from the academic medical center who worked with community-based organizations (CBOs) that provided HIV services in the Bronx invited representatives from the CBOs to participate as CAB members. At the time of this study, the CFAR CAB included 17 individuals who worked for CBOs. In addition, three to five faculty members from the academic medical center attended the quarterly meetings.

### Data collection

Between October and December 2012, we invited all 17 members of the CFAR CAB to participate in the study, using emailed study announcements and follow-up telephone calls. Individuals who consented to participate were interviewed at their offices or by phone. Reasons for declining participation were not systematically assessed. Interviews lasted 45 to 75 min and were audio-recorded and professionally transcribed. Participants were not compensated for their time. Following the interview, study participants were informed of the progress of the study via emails, phone calls and sharing of the preliminary manuscript.

Using open-ended questions, the interview guide focused on CAB members’: 1) HIV-related work and experience, 2) experiences with and attitudes about research and researchers, 3) evaluation of interactions with researchers, and 4) experiences on CABs presently and in the past. Interview questions were focused on CAB members’ experiences as representatives on an advisory board and not as community members themselves. The interview guide was revised iteratively to further explore common themes that emerged—for example about trust and power—after themes emerged in the first few interviews. The Albert Einstein College of Medicine's Institutional Review Board approved the study.

### Analysis

We used an inductive thematic analytic approach [32]. First, two authors (SS and JLS) reviewed the first ten interview transcripts and generated a list of categories and codes (open coding). The initial coding scheme was developed based on this list and refined to minimize overlap between codes. Using nVIVO qualitative data analysis software (QSR International Pty Ltd, Version 10), transcripts were iteratively coded by two independent coders, SS and either AB or LH. The study team and an interdisciplinary group of qualitative researchers reviewed each coded concept for internal consistency and examined the relationships between codes to identify thematic categories and themes. The coding scheme was iteratively revised to better identify distinct thematic content. Finally, all transcripts were independently coded by two authors (SS and AB or LH) using the final coding transcript. Discrepancies were resolved through consensus.

## Results

Fourteen of the 17 CAB members (82 %) participated in the study. Most (11) participants were non-Hispanic black or Hispanic, and nine were women. All had at least five years of experience in organizations that provided HIV-related services within the Bronx, NY. Four had graduate degrees (e.g. MPH or PhD). The community-based organizations (CBOs) that CAB members were affiliated with included harm reduction centers, peer education programs, housing agencies, and HIV advocacy groups. Eleven CAB members were current or former executive directors of a CBO. Reasons for joining the CAB included being asked to do so by research faculty, wanting to stay informed about HIV activities in the community, wanting to represent the community, and wanting to network with Bronx-based individuals working on HIV prevention and treatment efforts.

While CAB members reported some positive experiences with researchers, two prominent thematic categories that emerged were lack of trust in researchers and believing there was a power imbalance between CAB members and researchers. Subthemes within the category of lack of trust included: 1) researchers’ lack of respect for CBO interests and 2) researchers’ inadequate attention to building trust. Subthemes within the category of power imbalance were: 1) CAB members’ felt like inferior “token” members of the advisory board; 2) CAB members felt lack of communication with researchers led to disempowerment; and 3) CAB members held preconceived beliefs about researchers that led to perceived power imbalance. The third thematic category that emerged was suggestions for best practices to improve academic-community partnerships, which included: 1) using collaborations to build trust, 2) actively allocating power, and 3) sharing tangible research benefits with CBOs. Below, we present each thematic category and subtheme and describe CAB members’ perspectives on how these themes play out in academic-community partnerships, using exemplary quotes.

### Lack of trust

Based on previous experiences, CAB members reported feeling apprehensive about working with researchers. This “leeriness” weakened CAB members’ trust in their relationships with researchers. Drawing from these previous formative experiences, CAB members described the impact of researchers’ lack of investment in building trust with CAB members and community members.

#### Researchers’ lack of respect for CBO interests

Several CAB members described experiences that left them feeling distrustful of future interactions with researchers. For example, one CAB member who was an executive director of a CBO recounted an experience when a researcher had approached a low-level employee at the CBO to gain permission to recruit minors to participate in a research study about sexual health, rather than seeking approval directly from the organization’s leadership. Given the sensitive nature of the research, this CAB member felt the researcher had overstepped boundaries, and in so doing, had broken any trust that the agency could place in future researchers. The CAB member explained:

Researchers need to go to the head of the organization. They've got to understand [that] breakdown in communication can happen… That made us [the CBO] really rethink what we were doing [with research collaborations]. *Interview 9*

Other CAB members described experiences working with researchers who demonstrated lack of awareness about the needs of study participants. For example, one CAB member who is not the director of a CBO recalled a researcher who offered gift cards to a store that the organization’s participants could not access because travel to the closest store on public transportation would take two hours. For the CAB member, this decision represented a broader lack of understanding of study participants’ needs and therein created a feeling of discomfort in her attitude towards researchers:

[Researchers] have to be real about what the tradeoff is for our participants… I understand cash isn't always possible. But that kind of operating in a silo that a lot of researchers have… that lack of transparency of what the organization's getting out of participating or what the participants are getting out of participating, makes me feel very leery. *Interview 10*

Another CAB member who was an executive director of a CBO described having to wait over a year before awarded grant funds were dispensed from the academic center to the organization to pay staff. The CAB member stated, “It was like they just didn’t care …You [the academic center] are screwing your community group who should be building your future” *Interview 14*

#### Researchers’ inadequate attention to building trust

CAB members felt that researchers did not appreciate the importance of investing effort and time into building trust with CAB members or community members.

One CAB member who was an executive director of a CBO summarized the importance of trust-building as follows:

…the better you [researchers] use your community advisory boards and your community stakeholders,… the better the outcome. *Interview 6*

The same CAB member went on to highlight the importance of taking time to build trust:

Researchers can't be so fast to think that people are going to expose all their dirty laundry… There's a trust factor that has to be built and it takes time. *Interview 6*

Similarly, another CAB member who was a former executive director of the CBO explained that CBOs and their participants may be reluctant to work with researchers whom they do not know and trust:

I've had researchers approach me who I've never met, and it is a much more difficult and stressful process. A lot of the time, I am half-tempted to say no -- not because I don't believe in what they're trying to prove or investigate, but because I don't know that I can be sure that the participants are not going to be exploited. *Interview 11*

This quote highlights both the need and importance of the investment of time into trust building, and emphasizes that it is essential because CAB members may not naturally have faith in researchers and may fear ulterior motives such as being “exploited.”

### Power imbalance

CAB members also expressed strong views about the imbalance of power in relationships with researchers. CAB members described feeling like inferior “token” CAB members, explored the ways in which lack of communication led to disempowerment, and discussed the existence of a power imbalance.

#### Inferior status as inferior “token” CAB member

CAB members explored how the structure of advisory boards themselves gave more authority to researchers than to CAB members. One person wondered if CAB members had any real power, stating “As a member of an advisory board, sometimes I felt like a token” *Interview 15*. Another CAB member who was an executive director of a CBO described “serving” researchers and having little opportunity to challenge them.

You got picked to come to a meeting where they [the researchers] set the agenda. You nod and agree … There was no room for real engagement or articulation of difference with the community advisory board because you served at the mercy of the master… the academic center… that empanelled that group. *Interview 4*

The same CAB member expressed internal conflict about whether to challenge researchers and noted that doing so could interfere with future partnerships:

Do I become a rubber stamp professional? Or, do I really go out there and push the button and understand that people may not always like you and invite you to the party but I stood for something? *Interview 4*

#### Lack of communication causing disempowerment

CAB members felt that lack of communication contributed to a power imbalance in many academic-community partnerships. CAB members cited a lack of communication in the generation of research questions and in the dissemination of study findings. One CAB member explained the concern around not being involved in the study design phase:

Researchers come up with this idea and they come to you and say ‘we're doing this study, we'd like you to participate.’ What that really means is ‘we want access to your people… and say yes or no.’ … Come and have the conversation at the beginning, instead of researching stuff that you're kind of thinking is important in a vacuum… because what academics think is important is very different from what we think is important. *Interview 11*

CAB members also felt that the dissemination of study findings for studies in which they had participated was often inadequate. For example, this CAB member expressed that lack of sharing information fostered distrust between CAB members and researchers:

We don’t trust you [researchers]… Why? Because you came into my community. You wanted all this information. And then you turned around and hightailed it right out of my community. You didn't leave nothing behind. If you take something, you replace it… You want all this information from us and it made us feel like you… had your own agenda. *Interview 14*

Another CAB member explained how lack of sharing information reinforced an imbalance of power in the academic-community partnership:

There is no real quid pro quo in the relationship between academic centers and community people. You have people who come in with very good ideas about research and they do it and give no feedback. It's this top down view of research as being ‘we're going to do it on you and eventually we're going to come up with really good programs but in the process we're really not going to engage you.’ That's why the longer that [researchers] don't engage in a conversation, the more disempowered and the more the gap [persists] between researchers and community. *Interview 4*

#### Perceived power imbalance based on preconceived notions of researchers

Many CAB members believed that researchers held more power in the relationship than CBOs did. Some of these descriptions suggest that these notions were based on perceptions of power imbalance. For example, a CAB member who was an executive director of a CBO explained that sometimes the perception of power imbalance was created by CAB members’ own feeling of lack of confidence in working with researchers:

Many members in the communities do not feel confident enough because they feel that, ‘oh wow, these are doctors and researchers, they've got far more education than I do. They've been doing this research for years. I'm just poor little lowly me…just trying to make it to work every day.’ They don't feel confident. They don't feel that they are up to it. They don't feel that they have anything of value to offer. *Interview 7*

Similar to lack of confidence, another CAB member who was an executive director of a CBO commented that fear was a potential barrier to establishing an equitable relationship with researchers. S/he suggested that CAB members’ fear comes from a feeling of being unequally matched in their skills to researchers from “the ivory tower:” “[It’s the] fear of the unknown, fear of their own skillsets as agencies, fear of the ivory tower” *Interview 12*. S/he goes on to explain that this sense of unequal power affects how CAB and community members engage with researchers, resulting in their feeling more distanced from researchers and that researchers have more power than they do.

Both descriptions of fear of unequal skillsets and lack of confidence demonstrate that CAB members may have a perception of power imbalance even before encountering actual differences in power in working with researchers.

### Best practices

Having explored some of negative past experiences around trust and power, CAB members offered numerous examples of ways in which their concerns could be addressed and future experiences enriched. These suggestions included: 1) using collaboration to build trust, 2) actively allocating power, and 3) sharing tangible research benefits with CBOs.

#### Using collaboration to build trust

One suggestion that CAB members proposed to improve academic-community relationships was to increase bidirectional sharing of information and to increase CAB members’ participation in all stages of research. CAB members highlighted the importance of sharing information with the community as one way to improve academic-community partnerships. For example, a CAB member responded:

[Researchers] need to be able to engage [CAB members] and give them back the [study] results so that they can feel more empowered, so that they can develop trust, so that you can have a better working relationship between researchers and community because if you don't have that then we're going to continue in this vicious cycle. *Interview 4*

CAB members also wanted to be involved in forming the research question from the outset of the study. They recognized that the work needed to forge this type of bidirectional communication and collaboration would be difficult but emphasized that it would also be rewarding. One CAB member spoke of the potential benefits of improved involvement of CAB members in the research process: “I think that if we were part of the development of the studies, [researchers would] have a hell of a lot more buy-in” *Interview 10*

Similarly, another CAB member described the potential positive effects of an investment in building trusting partnerships:

It takes a lot of effort to engage and really build trust but when you do it, it's the best thing--it's the best research you're going to get because people will trust you. *Interview 7*

#### Actively allocating power

CAB members suggested explicit attention be paid to creating an environment of equitable power. One CAB member suggested that researchers acknowledge their position of power at the onset and commit to an equal partnership with CAB members:

There is no equality between a multimillion dollar academic health center and poor community folks. [Academic centers] always come in with this group having power. So you need to make sure that [researchers] understand that they're equal to us. That means that this group has to give up power. *Interview 4*

In keeping with the recommendation that researchers “give up power,” CAB members suggested restructuring of the CAB itself. For example, this CAB member proposed that the power imbalance could be addressed if CAB members were treated like members of a board of directors:

Community advisory boards usually empanel for a grant or for a proposal or something else…They have very limited power and very limited life beyond the term of the contract…With the board of directors we could fire the executive director. On a CAB we can't. On a CAB we can just give our opinion. But you should treat the CABs just the way you treat a board of directors… *Interview 4*

This CAB member suggests that by treating CAB members as boards of directors, the CAB’s impact would be increased because of a change in the power dynamics.

Another suggestion was that researchers and CABs share space—that rather than hold meetings in the research centers, researchers should plan on having CAB meetings in the community at well: “I think part of what needs to happen is that the academics come out to the community more often instead of us going to them all the time” *Interview 13* (*non executive director at CBO*). This suggestion is one example of a structural change that reflects CAB members’ desire to equalize the power dynamics between the community and researchers.

#### Sharing tangible research benefits with CBOs

CAB members suggested that researchers should be more deliberate in identifying and delivering tangible benefits for CBOs. One member explained that researchers’ focus must shift from concern about career advancement to finding lasting benefits for study participants:

Researchers should be thinking… ‘What's the policy implication?’ or ‘What's the program implication?’ …[apart from whether] you can publish it. Is that your priority? So you can keep your tenure? I don't care about your tenure and I don't want you to put my participants through surveys if they're not going to get anything out of it. *Interview 10*

One suggested way that researchers can provide benefit to a CBO was to help the CBO conduct studies to demonstrate the organization’s effectiveness:

We [CBOs] all know [our work is] effective, but you know it in your gut and you know it because you see it on the street, but that doesn't necessarily prove it to the powers that be. One of the things with [CBOs] is we should be seeking out researchers and academics and saying this is the stuff we need to prove. Help us prove it. *Interview 11*

Another suggestion to benefit CBOs was sharing of financial resources. One member asked that researchers hire and pay CBOs for their help with the study, as this financial incentive has a lasting impact on the sustainability of the organization. Several members recounted that one researcher had provided funds during implementation of a study and that this act made the research feel much more equitable.

## Discussion

This study demonstrates concerns about trust and power among individuals representing urban and minority communities in an HIV/AIDS CAB, and provides suggestions for best practices. In particular, CAB members reported that previous experiences with researchers led to a lack of trust in these relationships, and that academic-community partnerships were founded on and perpetuated researchers having more power. They suggested areas of improvement, or best practices, including having researchers directly address their concerns about trust and communication, giving CABs more power, and finding ways for research projects to benefit CBOs.

For CAB members in this study, lack of trust and unequal power dynamics were exemplified by communication failures. For example, buying unusable gift cards for participants or approaching the wrong individual in a CBO for study approval were two examples of poor communication. However, many of these researcher “mistakes” are likely to have been unintentional. This is consistent with previous studies that found that CAB members’ grievances were founded on unintended misunderstandings [16]. For example, case studies of a CAB paired with a state institution writing an HIV grant and another of eight CABs working with academic centers on HIV substance abuse found that initial difficulties CABs faced were around misinformation that led to misunderstandings between CAB members and researchers [7, 33].

In contrast to these studies, in our study, race may play a role in the unintentional nature of grievances ascribed to researchers, where the majority of CAB members and community members they represented were racial minorities. Some CAB members used terms that evoked the historical top-down nature of racial inequalities, including describing being on a CAB as “serving at the mercy of the master” or fearing being “exploited” by researchers. Furthermore, the impact of race can be seen in the discussion of power imbalance, where CAB members cited examples of perceived differences in power when discussing CAB members’ lack of confidence and fear of inadequate skillsets in working with academic researchers. These examples are subtle—they are not clear demonstrations of how researchers caused these feelings of inadequacies, rather these are feelings CAB members held prior to working with researchers. The subtle nature of these perceived power imbalances may indicate how CAB members’ experiences of overt racism or racial microagressions (defined as everyday acts that may be ambiguously interpreted as an insult or mistreatment) may affect their perception of power in working with researchers [34–36].

The themes of lack of trust and power imbalance that emerged in this study echo previous findings that researcher-CAB interactions can suffer from “outsider-insider tensions” [16], for example, when researchers are perceived to be outsiders looking in, misunderstanding and sometimes exploiting the insiders who are the focus of the research [21, 37, 38]. Furthermore, though this study focused on a CAB in New York City, similar themes of power and trust have been described outside of the United States [39–41], for example in South Africa where race also has important social implications [42, 43]. South Africa, with its post colonial, post-Apartheid racial tensions, offers similar examples of CABs which struggle from feelings of disempowerment, such as in Reddy et al.’s in-depth case study of a vaccine-related South African CAB where CAB members describe feeling “imposed upon” by their research partners [44]. Our study adds to this work by describing factors that influence the effectiveness of researcher-CAB partnerships focused on HIV/AIDS research in urban U.S. communities of racial and ethnic minorities. Our finding that unintentional misunderstandings or racial/ethnic differences can have a major impact on trust and perceived power imbalance in an urban HIV-focused CAB highlights how important it is for researchers to actively identify and address any misgivings CAB members have from the outset of the partnership.

CAB members in this study provided practical suggestions that are simple to implement and could markedly improve relationships with researchers. These included increasing communication and participation. For example, CAB members called for more involvement in the study design phase and for study findings to be shared with the participating CBO. Other suggestions, such as researchers sharing the benefits—financial or otherwise—with CBOs, support the theme of the need for increased CBO participation in the research process. This is consistent with James et al.’s study which noted that part of the CAB’s success was because it actively worked to avoid “drive-by research” [12]. Our study not only reinforces CAB members’ need to avoid “drive by” research, but differs in showing a desire among many CAB members to become more heavily involved in sharing the responsibilities of research. CAB members consistently emphasized the importance of an egalitarian partnership that would allow them to participate in all aspects of the study. While we recognize that this type of inclusive partnership may not be feasible in all research environments, researchers would still benefit from directly discussing expectations with CAB members at the study outset.

Our study supports evidence that while CABs can be a good mechanism to foster collaborative relationships with communities [11, 12], the presence of CABs alone is not enough to execute effective participatory research [7, 17]. Prior studies of successful CABs have identified tenets of success, which include exchanging information equally between researchers and CAB members, establishing expectations and limitations, and instilling a feedback mechanism to rectify grievances [1, 11, 14]. In our study, it is possible that the lack of these tenets for successful CABs accounted for some CAB members’ negative experiences. Notably, the CFAR CAB’s mission statement did not specify a goal to conduct participatory research that involves CAB members in each phase of research [31], as have other CAB mission statements that more closely exemplify CBPR methodology [40, 45]. Having researchers write a CAB mission statement together with CAB members may be another way to more fully align the needs of CAB members representing communities needs and researchers.

This study has several limitations. We focused on only one CAB, which limits generalizability to other advisory boards or institutions. However, our participants raised a number of broad themes that are likely to be relevant in other institutions situated in poor, urban communities involved in research focused on HIV/AIDS prevention and treatment. In addition, CAB members discussed experiences related to participating on several CABs, not experiences solely related to the CFAR CAB. Finally, we focused only on the experiences of CAB members; we did not examine the procedural workings of the CAB to understand its successes or failures from other perspectives, or the experiences of community members served by the CBOs represented on the CAB.

Our findings have three main implications. First, given the concerns about trust and power that many CAB members held before involvement in CABs, the fostering of trust and establishment of equanimity in power dynamics must be undertaken as an active process at the forefront of the academic-community partnership. Second, there are numerous opportunities for improvements in academic-community partnerships, many of which are straight-forward and simple to implement. Finally, the presence of CABs in HIV/AIDS research is not enough to ensure that good practices of CBPR are met, especially in communities of racial and ethnic minorities; rather, CABs must be framed around the tenets—such as exchanging information equally and establishing expectations at the forefront—that are foundational to successful participatory research.

## Conclusion

This study evaluated the relationship between Community Advisory Board (CAB) members and researchers involved in HIV/AIDS treatment and prevention research in an urban environment. Using qualitative evaluation, we found that trust and power affected relationships between CAB members and researchers, namely that CAB members’ previous experiences with researchers led to a lack of trust in these relationships, and that academic-community partnerships were founded on unequal power dynamics. CAB members also suggested areas for improvement via a listing of best practices (e.g. researchers directly address concerns about trust and communication; give CABs more power; ensure research projects directly benefit CBOs) and herein lies the impact of this study: It suggests that while historical tensions may exist between CAB members and researchers, there are multiple avenues to address and rectify these tensions and ensure future successful partnerships.

## Abbreviations

AIDS, acquired immune deficiency syndrome; CAB, community advisory board; CBO, community based organization; CBPR, community based participatory research; CFAR, Center for AIDS Research; ED, Executive Director; HIV, human immunodeficiency virus; NIAID, National Institute of Allergy and Infectious Disease
